# Posterior Percutaneous Screw Fixation to Treat Vertebral Fracture Non-union in Diffuse Idiopathic Skeletal Hyperostosis

**DOI:** 10.7759/cureus.19895

**Published:** 2021-11-25

**Authors:** Katherine E Wensley, Daniel Rolton

**Affiliations:** 1 Plastic and Reconstructive Surgery, Aberdeen Royal Infirmary, Aberdeen, GBR; 2 Trauma and Orthopaedics, Royal Berkshire National Health Service Foundation Trust, Reading, GBR

**Keywords:** spine imaging, minimally invasive and robotic spine surgery, vertebra fracture, ankylosing spinal disorder, diffuse idiopathic skeletal hyperostosis

## Abstract

Diffuse idiopathic skeletal hyperostosis (DISH) alters the biomechanical properties of the spine, rendering it highly prone to fracture, following even minor trauma. Risk of delayed diagnosis of vertebral fractures is particularly high in this cohort of patients since radiographs are notoriously difficult to interpret and presentation is late, due to difficulty distinguishing new from pre-existing back pain. Our case describes a gentleman in his late sixties with a six-month delay in presentation to our services with a T12 fracture, secondary to previously undiagnosed DISH, which had progressed to non-union. He underwent T9-L3 thoracolumbar posterior percutaneous stabilisation and fusion. At eighteen months follow-up, there was evidence of union, significant improvement in his pain, no focal neurology signs, and the patient had returned to his activities of daily living (ADLs).

## Introduction

Diffuse idiopathic skeletal hyperostosis (DISH) is an increasingly common non-inflammatory ankylosing spinal condition, often defined by the Resnick and Niwayama criteria, the hallmarks of which are: the bridging of four adjacent vertebral bodies by newly formed bone, without severe loss of the intervertebral disc height and an absence of degeneration of the apophyseal and sacroiliac joints [1].

These features result in progressive spinal fusion with reduced motion and kyphotic spinal column alignment. This alteration of spinal biomechanical properties results in a significantly increased fracture risk, in trauma considered otherwise minor [2]. Whilst fracture risk in DISH has not been quantified, in patients with ankylosing spondylitis-where fractures have been attributed to the same mechanism-risk [3,4] is increased fourfold [5]. In addition to this increased fracture risk, complications with healing are more prevalent and non-union rates higher. If vertebral fractures are unstable or progress to non-union, patients can be left with considerable pain and are at high risk of developing progressive neurological signs [6].

Incidence of delayed diagnosis of fractures in DISH is persistently high [7]. Multiple patient and doctor factors for these delays have been cited. These include late patient presentation due to difficulty distinguishing new from pre-existing back pain and difficulty interpreting radiographs due to fused joints and osteoporotic bone quality [8]. Clinician awareness of the increased fracture risk in DISH is therefore essential to facilitating CT or MRI screen scanning in this patient cohort. This allows timely diagnosis and appropriate management.

## Case presentation

We present the case of a gentleman in his late sixties who was referred to our spinal clinic by his general practitioner (GP). He first attended the clinic six months after a fall from standing height, complaining of ongoing back pain. Plain radiographs arranged by his GP three weeks after his fall revealed a T12 fracture, and over the next several months, his pain failed to settle. He had no radicular symptoms and no change in bowel or bladder habits. His medical history included a radical oesophagectomy for oesophageal carcinoma eight years previously, chronic obstructive pulmonary disease (COPD), asthma, and morbid obesity. He lived alone in a bungalow, normally mobilised without any walking aids and was independent with his activities of daily living (ADLs). Following the fracture, he occasionally used a stick to mobilise and reported that his pain was preventing him from carrying out his ADLs.

**Figure 1:**
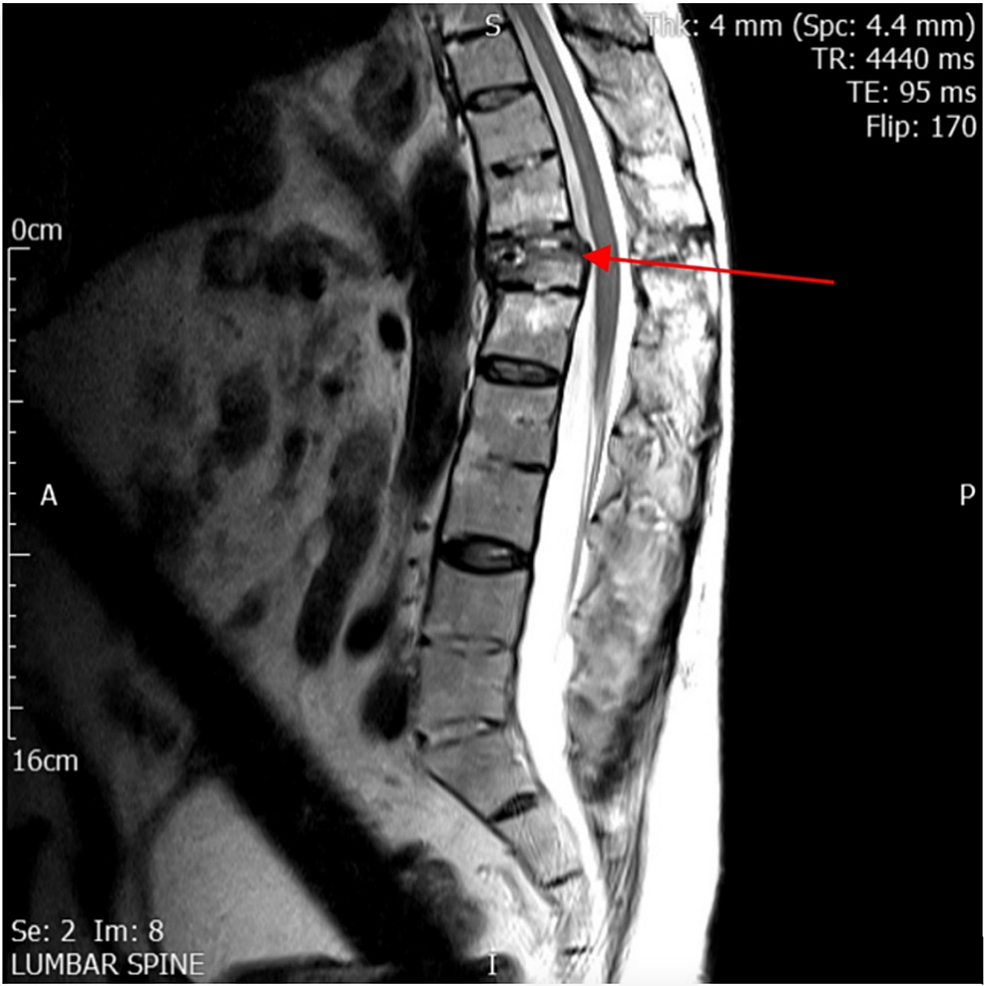
Sagittal T2 MRI image demonstrating T12 fracture non-union (red arrow), note that the image also demonstrates evidence of endplate changes.

Plain radiographs were arranged by his GP three weeks following his fall and revealed a minor wedge fracture of the T12 body with uncertain chronicity. Repeat imaging three months later suggested worsening of the fracture. Upon receipt of referral to our services, six months following his original injury, and prior to clinic attendance, he underwent an MRI scan on which there was evidence of fracture non-union (Figure 1).

**Figure 2:**
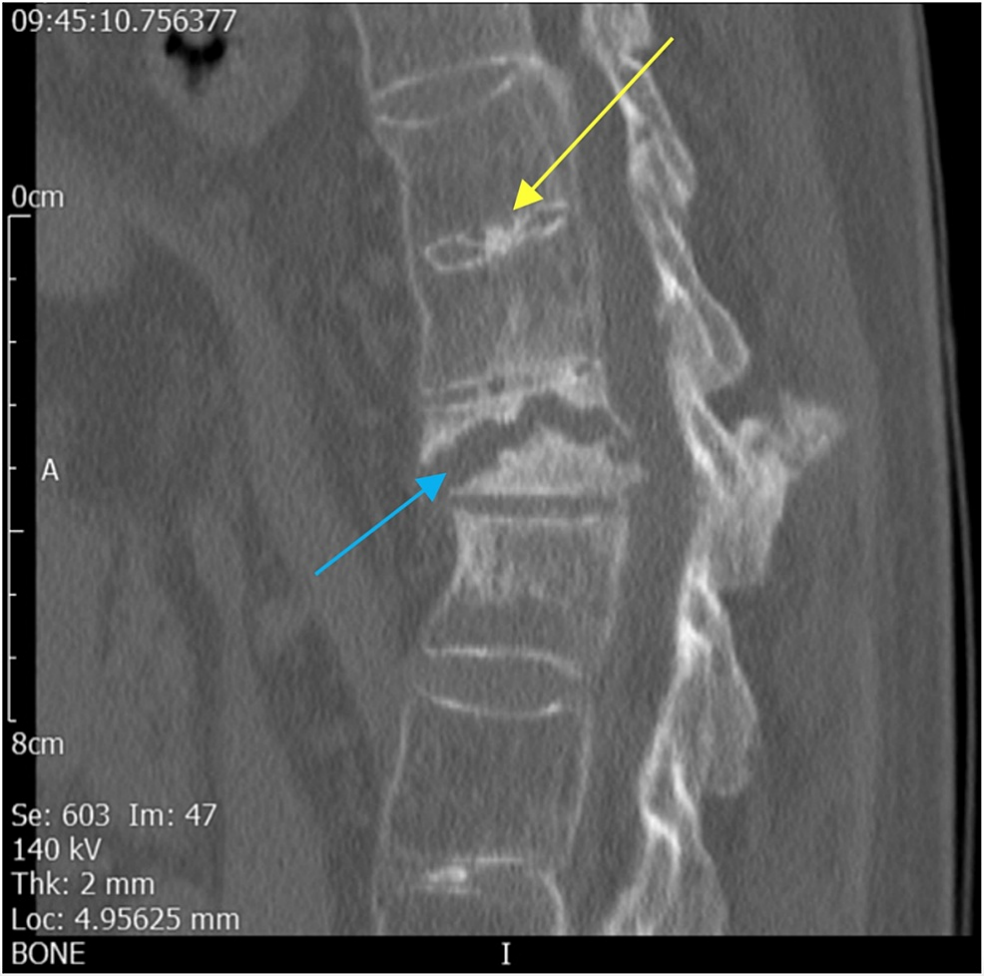
CT image demonstrating the horizontal T12 fracture to be irregular (blue arrow) with the reduction in vertebral body height, and no evidence of bony union. In keeping with the diagnosis of DISH, there is annulus fibrosus and central discal calcification (yellow arrow), associated with smooth flowing right anterolateral vertebral body osteophytosis. DISH: diffuse idiopathic skeletal hyperostosis.

Following assessment in the clinic, standing thoracolumbar radiographs and CT scan were arranged (Figure 2). The standing thoracolumbar radiographs were felt to be sub-optimal, but within the limitations of the study, the T12 fracture appeared to have worsened from initial radiographs, and with multi-level degenerative disc disease in the form of prominent anterior osteophyte formation was noted. On the basis of this imaging, it was felt that instrumentation was the best option to achieve adequate analgesia through spinal stabilisation and fracture union.

**Figure 3:**
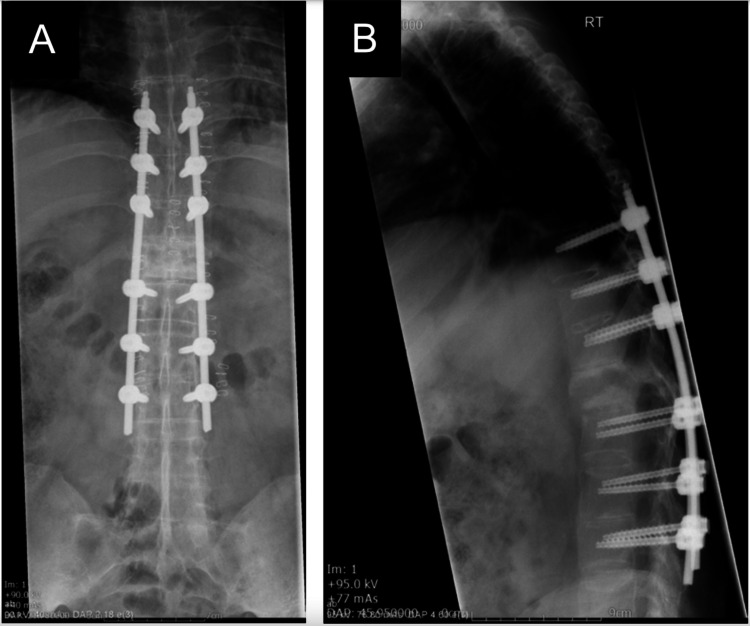
Post-operative radiographs (A) anterior-posterior view, (B) lateral view demonstrating good fracture reduction and satisfactory metalwork placement.

The procedure was performed under general anaesthetic by consultant spinal surgeon Daniel Rolton, with great care taken over patient positioning due to the risk of iatrogenic injury. Surgical stabilisation was achieved using a minimally invasive technique, through the use of posterior percutaneous pedicle screws, spanning three levels on either side of the fracture (from T9 to L3) and 5.5 mm titanium rods bilaterally. Pedicle screws were 6 mm × 40 mm for thoracic vertebrae and 6 mm × 45 mm for lumbar vertebrae (Viper, DePuy-Synthes). The operation was tolerated well and check imaging revealed satisfactory metalwork placement and satisfactory fracture reduction (Figures 3A, 3B). 

Our patient remained in hospital for two days post-operatively, with no complications. He was followed up in our clinic at four-month intervals from six weeks post-operatively to eighteen months post-operatively, at which point he was discharged back to the care of his GP. His surgical wounds healed well, with no metalwork complications, and there was radiological evidence of union. At four months post-operatively, he required no mobilisation aids and returned to work. When seen twelve months following his operation, he described himself as "85% better" compared to his pre-operative status, with no pain routinely, having discontinued his analgesia. However, he reported some backache when standing and walking for long periods.

## Discussion

DISH is a common but poorly understood condition. The aetiology is unknown but close associations with advancing age, diabetes, obesity, and a male sex have been demonstrated. The reported incidence varies widely based on age and population studied, from 2.9% in the Asian population [9] to 25% in the Caucasian population [10]. Given increasing life expectancies and increasing rates of type 2 diabetes mellitus and obesity, the incidence of DISH is likely to continue to rise in the coming years.

Non-operative management was previously favoured for vertebral fractures in patients with ankylosing spinal disease (ASD) but outcomes were generally poor [11,12]. Open pedicle screw fixation has also been widely practiced but more recently, both Yeoh et al. and Bredin et al. have reported good outcomes and lower complication rates with percutaneous pedicle screw fixation in patients with ASD [8,13]. However, to our knowledge, no series exist on the management of missed fractures that have progressed to non-union in patients with DISH.

As in this case, DISH patients are often heavily co-morbid, making minimally invasive surgery (MIS) a particularly attractive option [6,14,15]. A meta-analysis by McAnany et al. demonstrated equivalence in clinical outcomes between open and percutaneous procedures [16]. Additionally, recovery time, pain, and time until return to work have all been demonstrated to be reduced, when comparing percutaneous to open spinal procedures [17,18].

There is no consensus as to the optimal number of levels to fix in patients with ASD. The progressive fusion of the spinal column in DISH produces fractures similar to those seen in long bones, with a torque associated with the lever arms of the spine [14]. This, in combination with the reduced bone mineral density in DISH patients, necessitates multiple point fixation to stabilise the fracture. The decision to instrument three levels above and below the fracture was based on work by Werner et al. who suggest this as the minimum for effective fixation [19].

## Conclusions

With the ageing and increasingly co-morbid population, the prevalence of DISH is rising and should feature in the differential for patients presenting with back pain following minor trauma. Where fractures secondary to DISH are suspected, CT or MRI scanning should be considered as plain radiographs frequently fail to identify DISH. If the diagnosis is delayed and fractures have progressed to non-union, minimally invasive thoracolumbar posterior percutaneous stabilisation offers effective means of providing analgesia through the promotion of union.

Our case demonstrates the successful use of thoracolumbar posterior percutaneous stabilisation to manage a thoracic vertebral fracture, secondary to DISH, which had progressed to non-union. However, increased clinical awareness of DISH is vital to improve recognition rates of affected patients at initial presentation and as such, minimise the number of patients with a delayed diagnosis and fracture non-union.
